# Supplementary material for: Host generalists dominate fungal communities associated with alpine knotweed roots: a study of Sebacinales
Source: PeerJ. 2022 Oct 5;10:e14047. doi: 10.7717/peerj.14047 (PMC9547586; doi:10.7717/peerj.14047)
Supplement: Supplemental Information 1 — Sequences from the full dataset were subjected to hierarchical clustering to denser sampling of the dataset near the B. vivipara-related sequences and more sparse sampling elsewhere on the tree (see Supplementary Item 2 for a more detailed description of the clustering process). Because of the high number of originating countries and the great diversity of host taxa, the continents of the country of origin and the plant host classes were colour-coded and sequences from B. vivipara were marked with a distinct colour. [file peerj-10-14047-s001.pdf]

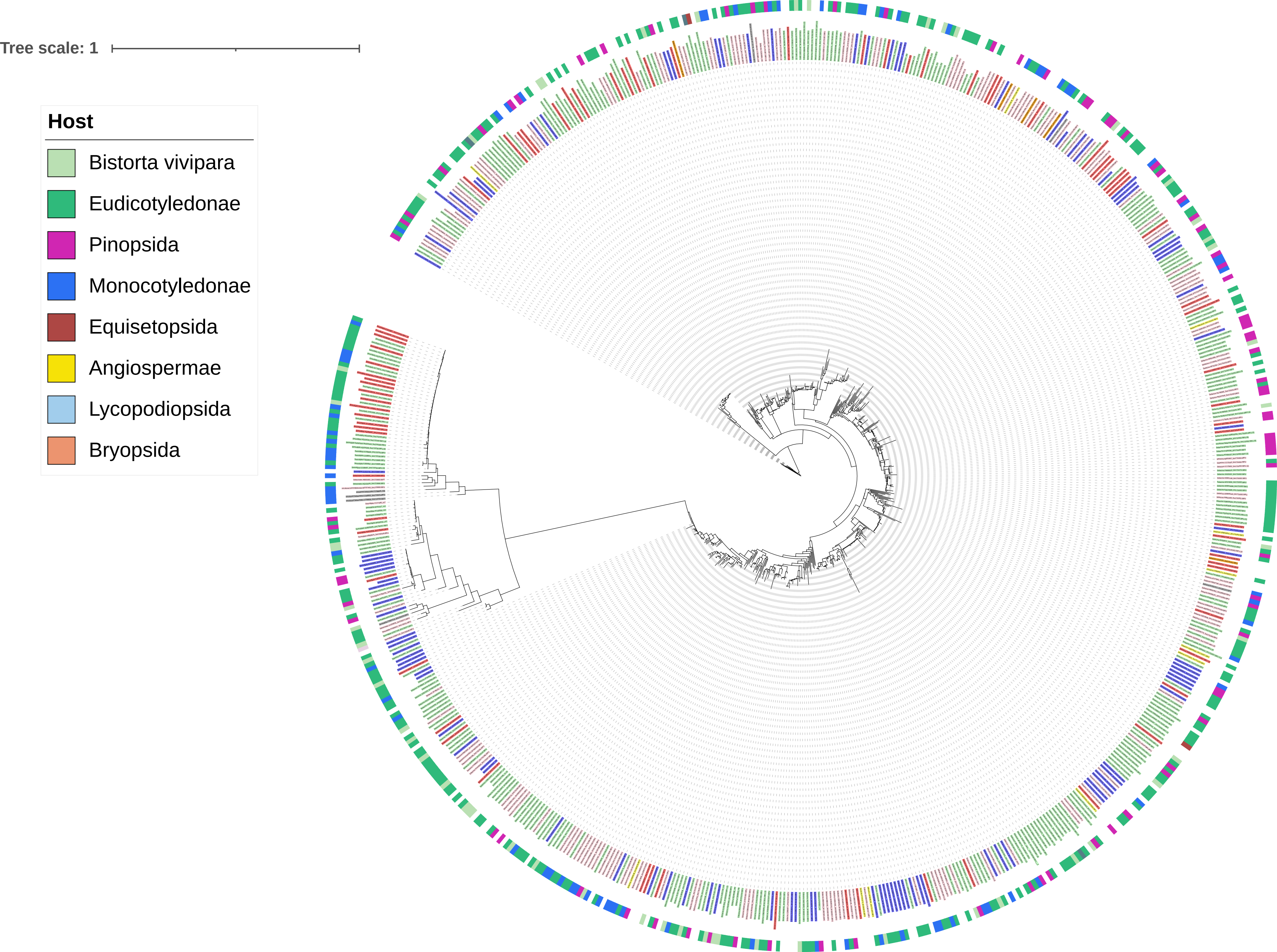
Tree scale: 1
Host
Bistorta vivipara
Eudicotyledonae
Pinopsida
Monocotyledonae
Equisetopsida
Angiospermae
Lycopodiopsida
Bryopsida
