# Supplementary figures and images for: Host generalists dominate fungal communities associated with alpine knotweed roots: a study of Sebacinales
Source: PeerJ. 2022 Oct 5;10:e14047. doi: 10.7717/peerj.14047 (PMC9547586; doi:10.7717/peerj.14047)

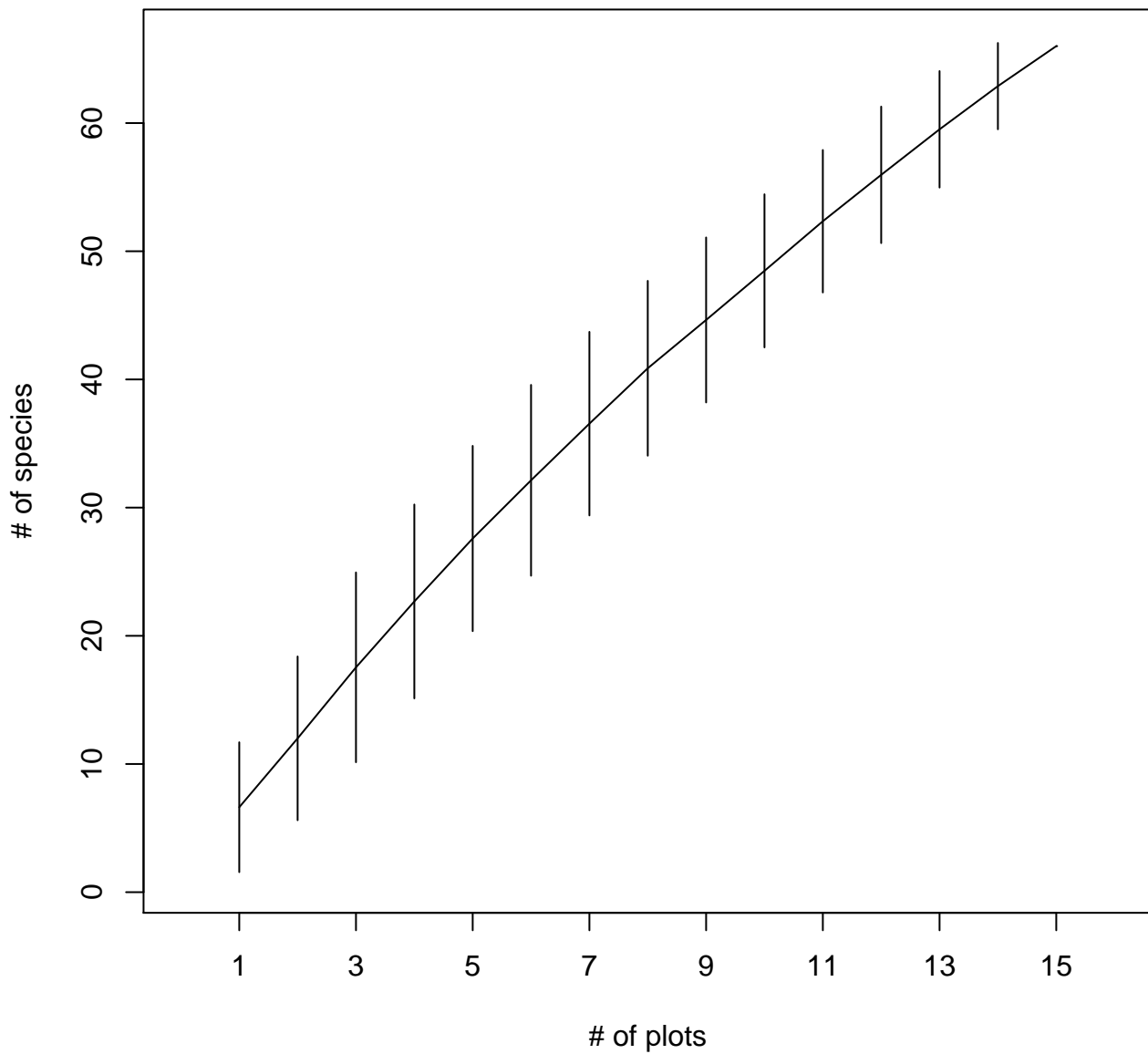

Supplement: Supplemental Information 2 [file peerj-10-14047-s002.pdf]
